# Feasibility and Effectiveness of Telephone-Based Telephysiotherapy for Treatment of Pain in Low-Resource Setting: A Retrospective Pre-Post Design

**DOI:** 10.1155/2020/2741278

**Published:** 2020-05-08

**Authors:** Shambhu P. Adhikari, Pragya Shrestha, Rubee Dev

**Affiliations:** ^1^Department of Physiotherapy, School of Medical Sciences, Kathmandu University, Dhulikhel, Nepal; ^2^Department of Physiotherapy and Rehabilitation, Star Hospital, Kathmandu, Nepal; ^3^Department of Nursing, School of Medical Sciences, Kathmandu University, Dhulikhel, Nepal

## Abstract

**Introduction:**

Telephysiotherapy (TPT) is a provision of physiotherapy services at a distance, using telecommunication technology when an in-person visit is not a feasible option. The objective of this study was to investigate the effectiveness of TPT in management of pain caused due to various problems among patients living in rural areas of a developing country.

**Methods:**

This retrospective study conducted in rural areas of Nepal recruited all patients who met the eligibility criteria during a day campaign. Two physiotherapists, one for assessment and another for treatment, were involved. Based on pretraining assessment finding, evidence-based individualized physiotherapy was prescribed. Pamphlets containing pictures of the prescribed exercises and instructions in the Nepali language were distributed. The treating physiotherapist made telephone calls to every patient each week to give necessary information, correction, modification, and progression of the exercise whatever required. At the end of the second and fourth weeks, pain was assessed using the Numeric Pain Rating Scale (NPRS) through telephone calls. The data were analyzed using ANOVA with repeated measures followed by pairwise comparisons.

**Results:**

Fifteen patients having pain due to various conditions participated in the study. The NPRS demonstrated significantly decreased pain (at rest: *F* = 3.5, *p* = 0.04, when worst: *F* = 26.4, *p* < 0.001, during activities of daily living: *F* = 16.6, *p* < 0.001, and during occupation: *F* = 15.6, *p* = 0.001) across time. The result met the minimal clinically important difference of NPRS, which is 2.

**Conclusions:**

The telephone-based TPT interventions demonstrated significant reduction in pain caused by various musculoskeletal problems. It could be a feasible and effective treatment option for the patients living in rural areas. However, we recommend for large-scale trials to establish effectiveness of the intervention and for its implication into routine clinical practice.

## 1. Introduction

Telephysiotherapy (TPT) is a provision of physiotherapy services at a distance, using telecommunication technology such as video conferencing or telephone meeting, when an in-person visit is not a feasible option [1]. TPT employs communication technologies either through an audio call or video call to facilitate the physiotherapy management of patients within their own homes. Clinically, TPT encompasses a range of rehabilitation and habilitation services that include assessment, monitoring, prevention, intervention, supervision, education, consultation, and counseling [2].

Patients who live far away from the clinics may find it difficult to attend the clinic regularly due to distance and cost of transportation [3]. In order to address these problems, TPT, which entails the use of telecommunications technology as a medium for providing information for therapeutic exercises to patients at homes that are at a distance from the physiotherapy clinics, should be considered [4]. Thus, TPT is a convenient way for patients to avoid long distance travelling and to perform basic physiotherapy exercises themselves at their own setting [5]. Beside this, physiotherapists could also monitor exercise parameters and provide necessary changes or progression through the TPT. The TPT not only saves time and money, but also improves quality of life of patients living in rural areas. In the absence of TPT, patients might have to discontinue physiotherapy treatment that may result into ineffective treatment and poor health outcome leading to deterioration of the quality of life (QoL) [6]. Though there might be many limitations and barriers of TPT, appropriate selection of the patients, interventions, and communication medium, supportive caregivers or health care personnel, and agreed schedule would help to make TPT an effective method [7].

In the context of developing countries like Nepal, TPT through Internet or video conference call is not possible due to feasibility issue as well as low literacy rate. So, simple telephone-based calls and understandable therapeutic materials (for example, printed flyers with figures and instructions) are the best means [8]. However, the therapeutic interventions have to be evidence-based with scientifically proven effectiveness [6, 7, 9]. Self-assessment and self-management are other key components that can be well integrated during TPT, which in turn increases exercise adherence and yields better clinical outcomes. The TPT also helps timely delivery of interventions with the removal of barriers (distance to clinic, transportation cost, travel time, and waiting list), flexible scheduling, and increased patient choice, resulting in a more patient-centered approach to care [7].

The TPT has already shown effectiveness in improving clinical outcomes in developed countries [7, 10, 11], but such studies are lacking in the context of developing countries where there are limited resources and the literacy rate is low. That is why it was important to conduct this study in the context of Nepal.

## 2. Materials and Methods

### 2.1. Study Design, Sample, and Setting

This was a retrospective study that included 15 participants suffering from pain due to prolapse intervertebral disc, tennis elbow, rheumatoid arthritis, and mechanical low back pain, traumatic ankle pain, and neck pain visiting Manekharkha and Bahunepati outreach centers, which are located in two rural municipalities of Nepal. These two centers are the outreach centers of the Dhulikhel Hospital, which were selected due to high need of physiotherapy services in their catchment areas. We included all patients from the 2-day camp who (a) visited outreach centers due to pain (≥1/10 in the Numerical Pain Rating Scale), (b) had those health conditions for which flyers or pamphlets in the Nepali language including pictures and instructions for physiotherapy treatment were available with our physiotherapists, (c) were educated (patients or primary caregivers who were able to read and follow the given instructions), (d) had mobile phone to reach them (patients or primary caregivers) and were able to communicate. Those who had hearing problems, severe comorbidities including depressive symptoms (based on medical/surgical reports or family member's response), and were not willing to give consent were excluded from the study. The mode of communication was through a mobile phone, the use of which has widely penetrated the rural settings, which is affordable, feasible, and has become an essential mode of telecommunication in Nepal [12].

### 2.2. Study Procedure

Informed written consent was obtained from the patient/caregiver for possible use of the data in research. Assessment was taken as part of the routine work. Two physiotherapists, one for assessment, called the assessor, and another for treatment, called the treatment provider, were involved.

### 2.3. Intervention

The preplanned treatment protocol for various conditions consisted of evidence-based basic physiotherapy techniques that were derived through the literature review, and it was previously applied for rehabilitation of postearthquake victims [13]. This protocol was published by Adhikari et al. in 2018 (principal investigator of the present study) and is freely available online (http://www.jfmpc.com/temp/JFamMedPrimaryCare761327-5192845_142528.pdf). The context and cultural background of the participants included in the postearthquake rehabilitation and in the present study was similar. Both were from the similar communities of the same district [13]. The intervention protocol also focused on psychosocial support besides management of pain-related impairments and activity restrictions [13, 14]. Individualized treatment was prescribed for four weeks based on patient-therapist collaboration. Pamphlets of each exercise with pictures and clear instructions in the Nepali language were provided to the participants [8]. Physiotherapist instructed the patients, their caregivers, and local health assistants on how to use the pamphlets based on the study by Azma et al. [11].

The physiotherapist who provided the treatment made telephone calls to each patient/caregiver every week (total four times in 4 weeks) and gave necessary information, correction, or modification of the prescribed exercises when needed. Progressive intervention was prescribed and provided when appropriate. Everything was guided according to the pamphlets that were provided to the patients and caregivers in advance. At times when the patient or caregiver could not understand any exercises or their progression, they were suggested to go to their nearby outreach center to take support from the health assistants who were also trained during the campaign at the first session. Nearby health assistants were trained with an aim to make the training program sustainable.

Telephone-based TPT was selected in this study because sophisticated communication media like real-time video conference and high-speed Internet are not widely available in developing countries [7]. Mobile telephone is a widely used telecommunication means in Nepal, and both literate as well as illiterate people living in rural areas can easily use it. Communication via a mobile telephone has also been documented to be an acceptable telehealth or telemonitoring system [15]. Flyers were provided to our participants, which could help as a guide during the exercise. This is consistent with the method applied in a study conducted in Nigeria [7]. Caregivers as well as local health assistants were trained together with the patients in this study so that one could help the other during need. Regular telephone calls were made as part of the TPT in this study. This was because physiotherapists' expertise remains critical to ensure that every component of the TPT protocol with their parameters are well aligned with the objectives and expected outcomes of the treatment [9]. We aimed to enhance the exercise adherence and compliance through regular calls made by the treatment provider [8, 16].

### 2.4. Outcome Measure

The Numeric Pain Rating Scale (NPRS) was selected as an outcome measure to access pain intensity. The NPRS is an 11-point scale from 0–10 where “0” = no pain and “10” = the most intense pain imaginable. In this scale, patients verbally select a value that is most in line with the intensity of pain that they have experienced in the last 24 hours. A written form is also frequently used with the numeric values of 0–10 written out. The NPRS has good sensitivity while producing data that can be statistically analyzed [17]. Excellent test-retest reliability and interrater reliability (*r* = 0.79–0.92) of this tool have been established with 100% agreement between two raters scoring the 0–10 point NPRS [18]. It has excellent concurrent validity with correlation between NPRS and the Visual Analogue Scale (*r* = 0.86) and correlation between NPRS and the Verbal Descriptor Scale (*r* = 0.88). NPRS has adequate construct validity correlation between NPRS and the Verbal Rating Scale (Spearman's *r* = 0.38) [18, 19]. In our study, NPRS was used to measure the pain level at rest, during worst, during activities of daily living (ADLs), during occupational work, and at stress. The NPRS can be administered verbally (in person and by telephone call as well) or graphically. The respondent is asked to indicate the numeric value on the segmented scale that best describes their pain intensity. Scores range from 0–10 points, with higher scores indicating greater pain intensity. This scale was selected because it is easy to use, is reliable and valid, and it takes short duration to administer [19, 20].

At the end of the second and fourth weeks, the patients were called through a mobile phone and the assessor evaluated pain using the Numeric Pain Rating Scale. During the campaign, patients, their caregivers, and health assistants of nearby outreach centers were instructed and well trained for scoring their pain in NPRS. At times, when patients had confusions, they used to seek help from caregivers or health assistants. So, we did not encounter any issues regarding administration of the outcome measure.

### 2.5. Data Analysis

Descriptive statistics were used to describe clinical and demographic characteristics of the participants. Since all the dependent variables were normally distributed (K-S test, *p* value > 0.05), the data were analyzed using one-way ANOVA with repeated measures. Mauchly's test was used to find the sphericity. Whenever sphericity was not met, the Greenhouse–Geisser factor was applied. Pairwise comparison was done, and the Bonferroni factor was applied to adjust for multiple comparisons. The significant level was considered at *p* value < 0.05. The data were analyzed using SPSS version 21.0.

## 3. Results

Participants with pain caused due to various conditions were included in this study. All 15 participants completed the protocol. Low back pain, either mechanical (20%) or due to prolapsed intervertebral disc (PIVD) (20%), was the major issue followed by knee, ankle, or elbow pain. Majority of the participants (53.3%) had pain between one and three months. All participants had musculoskeletal pain. All participants were from the adult age group. Both genders with age ranged between 36 and 70 years with the mean of 52.8 (SD: 11.56) participated in the study (Table 1).

The main occupation of all participants was farming. They used to work in the field for about 6–8 hours and do households work for about 2–3 hours per day. Children of 12 participants (80%) were living in the city or abroad for education or work. During this study, none of the participant reported to having medication specific to pain management. Long duration of work was an aggravating factor, and rest was the relieving factor for all the participants.

As depicted in Table 2, the repeated measures ANOVA with a Greenhouse–Geisser correction determined that mean NPRS for pain at rest differed significantly between three points of time (*F* = 3.5, *p*=0.04). Similarly, we found that mean NPRS for pain when worst (*F* = 26.4, *p* < 0.001), for pain during ADL (*F* = 16.6, *p* < 0.001), and pain during occupation (*F* = 15.6, *p*=0.001) all demonstrated significant difference between three points of time. When stress due to pain was measured in the form of NPRS, the mean score also demonstrated significant difference (*F* = 6.6, *p*=0.02) between three points of time.

The post hoc tests using the Bonferroni correction revealed that telephysiotherapy elicited significant reduction of pain when worst from pretraining to posttraining week 2 (*p*=0.02), pretraining to posttraining week 4 (*p* < 0.001), and posttraining week 2 to posttraining week 4 (*p*=0.009). Similarly, as shown in Table 3, the telephysiotherapy demonstrated significant reduction of pain during ADL as well as pain during occupation from pretraining to posttraining week 2, pretraining to posttraining week 4, and posttraining week 2 to posttraining week 4. However, post hoc tests did not reveal any significant changes on NPRS for pain at rest between any two points of time. Though, there was marked reduction of stress in the form of NPRS from pretraining to posttraining week 2 as well as pretraining to posttraining week 4, it was not statistically significant (*p*=0.06).

We had participants with the heterogeneous age group. When we analyzed in two age groups (<60 years versus ≥ 60 years), there was no significant time and age group interaction (*p*=0.05) on all continuous outcome variables.

## 4. Discussion

This study investigated the effect of a 4-week TPT intervention on pain management. The intervention demonstrated significant reduction of pain. The TPT intervention using a telephone call seemed to be a feasible option where high technology is beyond the reach of the people, and the literacy rate is low.

Our study demonstrated significant reduction on perceived pain “at rest,” “when worst,” “during ADL,” and “during occupation” after TPT. This finding was comparable with the findings of a meta-analysis in which pain level measured by the Visual Analogue Scale demonstrated better pain control with TPT [21]. This meta-analysis also reported that there was no any significant difference between TPT and face-to-face physiotherapy. Our study met minimal clinically important difference of 2 points in NPRS after 4 weeks of TPT on the variables; perceived pain “when worst (2.3),” “during ADL” (2.0), and “during occupation” (2.0) [22]. This indicated effectiveness of TPT for the pain management. A study by Multani et al., [23] also reported significant improvement in pain with 4 weeks of TPT program in patients with knee arthritis, which supports the findings of our study. Another study by Azma et al., also demonstrated significant pain reduction within the group across the time [11]. However, they measured the NPRS score 6 weeks after the treatment, which was longer in duration compared to our protocol.

A significant reduction of pain between pretraining to posttraining week 2 indicated that participants in this study demonstrated immediate pain relief with 2 weeks of TPT. A significant reduction between posttraining week 2 to posttraining week 4 indicated further reduction of pain with the continuation of the treatment. A study by Odole et al., demonstrated improvement in the physical domain with 4 weeks of TPT, which is consistent to our findings [7]. As per a clinical trial, reliance on TPT is higher compared to other forms of interventions for pain management for longer duration [24]. This is in line with our findings where participants demonstrated continued pain reduction from the second week to the fourth week. This could be because of better adherence in their own setting [14].

The intervention in our study was also focused on psychosocial support besides management of pain-related impairments and activity restrictions. This could have yielded significant reduction on pain-related stress among the participants in our study. The continued reduction of pain until 4 weeks could be due to continuous engagement of the participants in exercise. Therefore, TPT may encourage patients to be proactive for self-analysis and self-encouragement to engage with the exercises. This explanation is consistent with the hypothesis given by Knudsen et al., in a telerehabilitation study on cardiac patients [14] where the authors stated that the better improvement with the quality of life in the telerehabilitation group could be because of patients' increased activation.

Based on Qaseem et al.'s classification, acute (<1 month) or subacute (≥1 month to 3 months) or chronic (>3 months) [25], the majority of our participants had subacute pain. The duration of pain in the previous study has not been well described [7, 23]. Mean age, gender, and pretraining pain intensity of our participants were comparable with the characteristics of the participants of other studies [7, 11]. Since there was no significant time and age group interaction, there might not be an influence of age in the participants included in the present study.

Though we did not measure the multidimensional aspect of the pain, the religious, environmental, cognitive, and neurophysiological aspects might not have much effect, as all the participants were from the same community and had similar contexts and background. Furthermore, our objective was to explore the feasibility and outcome in general and convert the routine work into research. So, measuring many aspects of pain was beyond the scope of this study. To minimize influence of various multidimensional factors of pain, we had excluded participants with depressive symptoms, counseling session was integrated in the treatment session, and all interventions were selected in patient-therapist collaboration. However, it is important to examine the effect of multidimensional factors on pain management through telephysiotherapy in future studies.

### 4.1. Strengths and Limitations

TPT is advancing rapidly in developed countries where patients are digitally connected [9]. To our knowledge, this is a first study to address pain on heterogeneous participants (with respect to cause and duration of pain) through TPT, which could be a choice of delivery in the developing countries because older population is rapidly increasing who are in increased need of home-based rehabilitation for enhancing their wellness and QoL. We did in-person evaluation of every individual during the community campaign held at the outreach centers. Based on the evaluation, the first session of in-person physiotherapy treatment was administered to them. The purpose of the in-person first session was to avoid the possible risk of injury [26], to orient them about the exercise and progression plan, and to train them on how to follow instructions provided in the pamphlets which is a methodological strength of this study.

Our study also has some limitations. First, this retrospective study was conducted in a relatively small sample without a control group for comparison. Second, we could not measure exercise adherence. Variation in exercise adherence might have affected the result because patient perspectives and preference may differ between TPT versus face-to-face physiotherapy. Finally, there were no comparable published data on the effect of TPT among individuals with pain due to similar conditions we included in our cultural context and background. Therefore, comparisons were made on the findings of this study with the similar studies.

## 5. Conclusions

Telephone-based TPT intervention demonstrated significant reduction in pain caused by various musculoskeletal problems. Therefore, TPT is feasible and helpful in management of pain due to various musculoskeletal conditions in individuals living in rural areas who have barriers to accessing health centers daily. The findings of our study would be applicable in low-resource countries like Nepal. We recommend large-scale randomized controlled trials for future research to establish effectiveness of the intervention and its implication into routine clinical practice.

## Figures and Tables

**Table 1:** Demographic and clinical characteristics of the participants (*N* = 15).

Code	Age (year)	Gender	Pain duration	Diagnosis
01	56	Female	Acute	Low back pain (mechanical)
02	61	Female	Subacute	Prolapse intervertebral disc
03	71	Male	Subacute	Rheumatoid arthritis
04	57	Male	Chronic	Coccydynia
05	60	Female	Subacute	Tennis elbow
06	63	Male	Acute	Traumatic ankle pain
07	40	Male	Subacute	Tennis elbow
08	41	Male	Acute	Neck pain
09	50	Female	Acute	Low back pain
10	40	Female	Subacute	Low back pain
11	36	Female	Subacute	Prolapse intervertebral disc
12	40	Female	Chronic	OA knee
13	60	Female	Subacute	Traumatic ankle pain
14	70	Male	Chronic	OA knee
15	48	Female	Subacute	Prolapse intervertebral disc
Mean (SD)/number (%)	Mean (SD): 52.8 (11.56)	Male: 6 (40%)	Acute: 4 (26.7%)	Low back pain: 3 (20%)
		Female: 9 (60%)	Subacute: 8 (53.3%)	PIVD: 3 (20%)
			Chronic: 3 (20%)	Rheumatoid arthritis: 1 (6.7%)
				Coccydynia: 1 (6.7%)
				Tennis elbow: 2 (13.3%)
				Traumatic ankle pain: 2 (13.3%)
				Neck pain: 1 (6.7%)
				OA knee: 2 (13.3%)

*Note.* PIVD: prolapse intervertebral disc, OA: osteoarthritis, and SD: standard deviation.

**Table 2:** Outcome of the ANOVA with repeated measures on different variables (*N* = 15).

Variables	Mean (SD)			F	*p* value	Effect size
	Pretraining	Posttraining week 2	Posttraining week 4			
NPRS for pain at rest	2.93 (2.7)	2.66 (2.5)	2.6 (2.5)	3.5	0.04^*∗*^	0.2
NPRS for pain when worst	8.6 (1.7)	7.1 (2.1)	6.3 (1.9)	26.4	<0.001^*∗*^	0.7
NPRS for pain during ADL	7.7 (3.0)	6.4 (2.8)	5.7 (2.7)	16.6	<0.001^*∗*^	0.5
NPRS for pain during occupation	8.4 (1.9)	7.0 (2.2)	6.4 (2.3)	15.6	0.001^*∗*^	0.5
Stress due to pain in the form of NPRS	7.3 (2.3)	6.2 (2.3)	6.2 (2.3)	6.6	0.02^*∗*^	0.3

*Note.* ^*∗*^Indicates significance at *p* value < 0.05, SD: standard deviation, ADL: activities of daily living, ANOVA: analysis of variance, N: number of participants, and NPRS: Numerical Pain Rating Scale.

**Table 3:** Multiple comparisons between three points of time with Bonferroni adjustment.

Variables	Time	*p* values		
		Pretraining	Posttraining week 2	Posttraining week 4
NPRS for pain at rest	Pretraining	—	0.3	0.1
	Posttraining week 2	0.3	—	1.0
	Posttraining week 4	0.1	1.0	—
NPRS for pain when worst	Pretraining	—	0.002^*∗*^	<0.001^*∗*^
	Posttraining week 2	0.002^*∗*^	—	0.009^*∗*^
	Posttraining week 4	<0.001^*∗*^	0.009^*∗*^	—
NPRS for pain during ADL	Pretraining	—	0.01^*∗*^	0.001^*∗*^
	Posttraining week 2	0.01^*∗*^	—	0.009^*∗*^
	Posttraining week 4	0.001^*∗*^	0.009^*∗*^	—
NPRS for pain during occupation	Pretraining	—	0.006^*∗*^	0.003^*∗*^
	Posttraining week 2	0.006^*∗*^	—	0.04^*∗*^
	Posttraining week 4	0.003^*∗*^	0.04^*∗*^	—
Stress due to pain in the form of NPRS	Pretraining	—	0.06	0.06
	Posttraining week 2	0.06	—	1.00
	Posttraining week 4	0.06	1.00	—

*Note.* ^*∗*^Indicates significance at *p* value < 0.05, ADL: activities of daily living, and NPRS: Numeric Pain Rating Scale.

## Data Availability

The data used to support the findings of this study are available from the corresponding author upon request.
